# Temperature alters the toxicological impacts of plant terpenoids on the polyphagous model herbivore *Vanessa cardui*

**DOI:** 10.1007/s10886-023-01449-8

**Published:** 2023-09-11

**Authors:** Mari R. Irving, Eric W. Goolsby, Hannah Stanford, Simone Lim-Hing, Maria Urrea, Chase M. Mason

**Affiliations:** 1 Department of Biology, University of Central Florida, Orlando, FL 32816 USA; 2 Department of Ecology, Evolution, and Marine Biology, University of California Santa Barbara, Santa Barbara, CA 93106 USA; 3 Department of Plant Biology, University of Georgia, Athens, GA 30602 USA

**Keywords:** Herbivory, Insect development, Lepidoptera, LC50, Plant defense

## Abstract

Terpenes are a major class of secondary metabolites present in all plants, and long hypothesized to have diversified in response to specific plant-herbivore interactions. Herbivory is a major biotic interaction that plays out across broad temporal and spatial scales that vary dramatically in temperature regimes, both due to climatic variation across geographic locations as well as the effect of seasonality. In addition, there is an emerging understanding that global climate change will continue to alter the temperature regimes of nearly every habitat on Earth over the coming centuries. Regardless of source, variation in temperature may influence herbivory, in particular via changes in the efficacy and impacts of plant defensive chemistry. This study aims to characterize temperature-driven variation in toxicological effects across several structural classes of terpenes in the model herbivore *Vanessa cardui*, the painted lady butterfly. We observed a general increase in monoterpene toxicity to larvae, pupa, and adults at higher temperatures, as well as an increase in development time as terpene concentration increased. Results obtained from this study yield insights into possible drivers of seasonal variation in plant terpene production as well as inform effects of rising global temperatures on plant-insect interactions. In the context of other known effects of climate change on plant-herbivore interactions like carbon fertilization and compensatory feeding, temperature-driven changes in plant chemical defense efficacy may further complicate the prediction of climate change impacts on the fundamental ecological process of herbivory.

**Supplementary Information:**

The online version contains supplementary material available at 10.1007/s10886-023-01449-8.

## Introduction

To deter herbivory, plants possess sophisticated and diverse arsenals of chemical defenses composed of many individual secondary metabolites. Plant secondary metabolites are ubiquitous in the plant kingdom and have a wide range of ecological roles such as mediating symbiotic interactions and plant-plant signaling, direct defense against herbivores and pathogens, and providing protection against abiotic stressors such as frost and drought (Dixon and Paiva 1995; Gershenzon and Dudareva 2012). Chemical defenses can be produced constitutively or induced upon attack to directly and indirectly thwart herbivore attacks by exerting toxic effects upon contact or ingestion as well as by attracting natural predators of herbivores (Howe and Jander 2008).

One major class of secondary metabolites are the terpenes, which have exceptional abundance and diversity in flowering plants. The majority of the diversity in terpenoids is lineage specific - of the 25,000 terpenes reported, only about 100 are present across all plant species (Tissier et al. 2014). An explanation for the abundance of lineage specific compounds is the evolutionary arms race proposed by Ehrlich and Raven (1964) in which existing molecules are modified to create novel compounds unexperienced by herbivores. The most well-established function for terpenes is defense against biological enemies through direct and indirect defense, but assigning specific roles is difficult to establish because multiple terpenes are often expressed at once in volatile bouquets (Pichersky and Raguso 2018). For those with direct roles in chemical defense against herbivores, terpenes have been shown to decrease the likelihood of survival to reproduction and slow the rate of larval growth (Kumbasli and Bauce 2013). In lepidopteran larvae, terpenes block the stimulatory effects of glucose and inositol on chemosensory receptor cells located on the mouthparts (Gershenzon and Dudareva 2012). Several monoterpenoids, including linalool and limonene, may be neurotoxic or act as insect growth regulators, disrupting the normal process of morphogenesis (Balandrin and Klocke 1988; Coats et al. 1991). Leaf allelochemical composition, including terpene expression, is heavily influenced by both biotic and abiotic conditions (Kleiner 1989; Hunter 1992), and recent studies suggest that seasonality may regulate the production of a variety of defense compounds (Gols et al. 2018; Blanchard and Bowers 2020).

Although the extent to which seasonal changes such as daylength and temperature influence plant defenses remains elusive, their impacts on insects are extensively studied. Seasonal fluctuations in daylength, temperature, and host plant availability influence the metabolic activities of many short-lived herbivores, including insects. Much of insect life history revolves around favorable and unfavorable conditions for growth and reproduction, which many species rely on seasonal cues to detect. Developmental tactics such as diapause or dispersal via migration are strategies to avoid energy expenditures during unfavorable periods. Diapause permits insect survival under adverse climatic conditions and synchronizes the life cycles of individual insects within a population as well as with the phenology of their food supply (Chippendale 1982), and migration can permit insect species to continue to avoid adverse climatic conditions or a lack of food availability altogether (Hahn and Denlinger 2011, Chapman et al. 2015, Hu et al. 2021). Insects are cued to initiate diapause or migration when daylength, moisture, temperature, and foliage quality decline at the end of growing seasons. For example, the end of the monarch breeding season and beginning of their migration to southern overwintering grounds is signaled by the dormancy of their larval host plant, milkweed (Goehring and Oberhauser 2002). In many ecosystems, it is typical for plant phenology to temporally restrict the growth and development of specific phytophagous insects (Feeny 1970; Wint 1983).

The extensive history of coevolution between plants and insects has created seasonally-regulated synchronously-timed life events within communities, such as insect emergence being aligned with host leaf-out phenology, but global climate change is complicating these sensitive dynamics by altering the geographical ranges, population dynamics, and phenologies of many organisms (Bale et al. 2002). Desynchrony between emergence or hatching and host plant phenology can have disastrous and long-lasting impacts on both plant and herbivore populations (Yukawa and Akimoto 2006; Teder 2020). For herbivorous insects, emergence before sufficient host plant availability can result in intensive competition and mass mortality events (Forrest 2016; Fuentealba et al. 2017). Likewise, an increase in ambient temperature can have dramatic effects on developmental rates, reproductive potential, overwintering survival, and the number of generations occurring within a season (Ayres and Lombardero 2000). Global warming has created prolonged reproductive windows, which in turn can bolster insect populations (Stoeckli et al. 2012).

Larger herbivore populations present for a longer portion of the year paired with declining leaf nutrient quality will potentially increase the length and degree of herbivory pressure on plant populations (Marini et al. 2017). Assessment of herbarium specimens over the past century suggests that increasing temperatures have increased rates of herbivory in temperate plants, in parallel with increases in the predicted occurrence of many dozens of species of lepidopteran herbivores (Meineke et al. 2019). Multiple generations of herbivores can inflict unprecedented levels of damage over the course of a single season, particularly on long-lived plants such as pines (Porter et al. 1991). As global temperatures rise, instances of altered voltinism have already been observed in herbivorous insects. In one such study of 263 multivoltine central European lepidopteran species spanning the last 40 years, over half of species displayed an increased frequency of intra-year second and subsequent generations, and a quarter of species displayed constant increases in the number of generations per year (Altermatt 2010). Increased herbivore voltinism not only poses a substantial threat to the biodiversity of plant communities but also has broad implications for agricultural losses from pests (Hamann et al. 2021). In the case of the rice leaffolder (*Cnaphalocrosis medinalis*), warmer temperatures have been associated with increased outbreaks, which reduces rice yield potential and increases management costs (Ali et al. 2019). Destruction potential is further heightened as plant nutritional quality declines. Nitrogen, the primary nutritional element limiting insect growth, is known to be present at subaverage concentrations in foliage subject to elevated CO_2_ and temperature – which in turn stimulates insects to consume more foliage to compensate for reduced nutrient availability (Zvereva and Kozlov 2006)_._ The evidence to date suggests that in the future recurring herbivore outbreaks are likely to become more frequent, and baseline herbivore pressure more destructive, due to temperature-associated developmental acceleration, increases in voltinism, and feeding stimulation.

As rising global temperatures increase the rate of development of insect herbivores, plant defensive secondary metabolites could directly counter this effect by inducing insect developmental delays. Likewise, feeding stimulation may be countered by increased rates of insect mortality upon ingestion of relatively larger doses of chemical defenses per unit of nutrients obtained. However, these counteracting effects may be undermined if the efficacy of chemical defenses against insect herbivores is also altered by temperature, for instance due to temperature effects on insect metabolism. As the majority of primary productivity enters food webs in terrestrial ecosystems through herbivory, understanding how the efficacy of plant chemical defenses will change with ambient temperature is key to making informed predictions about the response of plant-insect interactions to climate change.

To test the hypothesis that the efficacy of chemical defense is altered by temperature, we assess the temperature-dependent impacts of four common terpene defenses in the model insect herbivore *Vanessa cardui* (Lepidoptera: Nymphalidae) – known as the thistle caterpillar or painted lady butterfly. This is one of the most widely distributed butterflies in the world, absent only from South America and Antarctica. The duration of the life cycle ranges from 21 days to multiple months, driven by variation across its near-cosmopolitan distribution, as well as its migratory behavior, and breadth of diet. *Vanessa cardui* is highly polyphagous, feeding on host species spanning at least ten different plant orders (Celorio-Mancera 2016), and a serious agricultural pest of crops as diverse as corn, alfalfa, sunflowers, beans, and soy (Williams 1970). This species has also historically exhibited large fluctuations in population size over time and mass migrations of millions of individuals (Kelly and Debinski 1999; Hu et al. 2021), making the likelihood of rapid increases in insect abundance and resulting herbivory pressure likely.

Overall, this study asks two questions: (1) how does temperature alter the toxicity of terpene defenses to *Vanessa cardui* in terms of mortality and insect mass, and (2) how do temperature and dietary terpene concentration affect the rate of *Vanessa cardui* development?

## Methods

### Experimental design

In order to assess how temperature alters the efficacy of terpene defenses, controlled experiments were performed manipulating both temperature and dietary terpene concentration and recording mortality, adult mass, and developmental responses of *V. cardui*. In addition to its relevant ecology, this species is easy to rear in laboratory settings and thrives on general lepidopteran diets (Ahmad et al. 1989). Individual insects were reared from eggs (Carolina Biological Supply Company, Burlington, NC), in individual 1.25-ounce plastic cups (Frontier Agricultural Sciences, Newark, DE) on optimized artificial *V. cardui* diet (Frontier Agricultural Sciences, Newark, DE). We chose commercially sourced *V. cardui* to avoid issues such as presence of disease and temporally-limited abundance that are often experienced when using field-sourced specimens (Kelly and Debinski 1999). Rearing insects, including *V. cardui,* on artificial diet is common practice in a broad range of applications such as transcriptomics, pest management, and biocontrol studies (Connahs et al. 2016; Sørensen et al. 2012; Li et al. 2014). Artificial diet was adulterated with one of four selected terpenes at ten concentrations including a control with no terpenes present (Datasets S1-S3), as informed by preliminary pilot trials for each compound conducted at room temperature. To adequately represent a cross-section of terpene diversity, four botanically common terpenoids belonging to different structural subclasses were tested: D-limonene (cyclic monoterpene, CAS# 5989-27-5, concentrations from 0-12%), 1,8-cineole (cyclic monoterpene ether, CAS# 470-82-6, concentrations from 0-9%), linalool (acyclic monoterpene alcohol, CAS# 78-70-6, concentrations from 0-2.5%), and beta-caryophyllene (bicyclic sesquiterpene, CAS# 87-44-5, concentrations from 0-6%). Concentrations differed among the four terpenes to adequately span sublethal to lethal concentrations, and the concentrations used for each terpene were not kept identical between trials, as multiple uninformative high doses with full mortality in early trials were replaced in later trials with additional intermediate doses for higher precision of estimated toxicity (see Dataset S1-S3). The ranges of concentrations used in this study span reported plant tissue concentrations for terpenoids generally (Blanch et al. 2009; Kopaczyk et al. 2020), and for these terpenoids in particular (King et al. 2006; Mewalal et al. 2017; Song et al. 2019; Saunier et al. 2022).

Terpenoids were purchased from Fisher Scientific (Waltham, MA) as liquids of high percent purity (96-99%) and homogenized with diet media in a blender. Diet was prepared 4-7 days prior to insect placement, with 0.5 liters prepared per treatment dose and approximately 17 grams distributed into each replicate rearing cup (slightly over half of the cup volume). Prepared cups were then stored at 4°C with sealed lids to prevent terpene loss due to volatilization. Insects were reared in climate-controlled incubators (136LL, Percival, Inc) for the duration of their life cycle at a L12:D12 photoperiod and at one of three different temperatures, 24°C, 27°C, or 30°C, to represent the lower threshold, median temperature, and upper threshold at which *V. cardui* is able to successfully complete its full life cycle (Pakyari et al. 2019; Poston et al. 1977). A single trial run consisted of a total of 400 individuals - four terpenes at ten concentrations with ten replicate insects at each concentration – reared from egg to adult at a single temperature. Two replicate trial runs were performed at each temperature, for a total of six trial runs overall. The focal response variables were rate of hatching failure, larval mortality, pupa mortality, adult mortality, development time from hatching to pupation and emergence (eclosure), and adult dry biomass.

### Specimen rearing and scoring procedures

Specimens were reared from eggs, following typical insect ecotoxicology procedures. For each diet containing cup, three eggs were placed onto wax paper to separate them from direct contact with the surface of the diet, otherwise diet media would interfere with the outer egg membrane and hatching could not occur. A two-ply tissue paper was placed between the cup opening and lid as an attachment point for the chrysalis cremaster at pupation. Approximately eight small airholes were made on the lid of each cup to permit gas exchange. These cups were then placed into their respective growth chambers, using a randomized spatial design to assign individual larvae to shelves. Beginning the second week of the trial, 40 μl of water was added to the surface of diets each week to maintain optimum diet moisture. Once hatching occurred (typically one to two days) additional larvae were removed along with the residing wax paper so only one out of the three original caterpillars remained in each cup. For diet treatments that fully inhibited hatching within a cup, additional larvae that had been reared on control diet in parallel to experimental specimens were used as replacements for the absent larvae, to assess terpene toxicity in the following development stages. Small paintbrushes were used to transfer these replacement larvae onto the terpene treated diets that inhibited hatching, occurring approximately three days after hatching, depending on temperature, when they were robust enough to tolerate manipulation. Once chrysalis formation occurred for a replicate insect, the tissue paper upon which the cremaster was secured was pinned to the top of a standard 28 L netted flight box to permit completion of the life cycle to adult emergence. Similar to larval cups, flight boxes were placed into their respective incubators and assigned to shelves using a randomized spatial design. Since space in growth chambers was limited and each pupa could not be allotted their own individual flight box, specimens were grouped by the specific treatment diet on which they were reared (by concentration within each compound).

Mortality and stage of development were recorded at least every other day. The date of hatching commenced development tracking and was considered day 0 when counting days till the next development stage was reached. The date of chrysalis formation marked the transition from larval to pupal phase, and date of emergence marked the transition from pupal phase into adulthood. Individual specimens were reared alone in a single container from egg to pupa so scoring data could be assigned to those individuals with certainty, whereas days to adulthood could only be estimated per specimen because pupa were transferred to flight chambers with others from their treatment. In cases where only one adult emerged from its chrysalis during a scoring interval (one to two days), days to adulthood could be known with certainty. In cases where multiple adults emerged during a scoring interval, days to adulthood could be estimated to within the scoring interval (one to two days). In cases where a pupa did not form any or only a weak cremaster, they were placed on the floor of the flight chamber and recorded as an improperly formed chrysalis. The development of these pupa could be easily tracked into adulthood because if they emerged, they were typically deformed and stuck in place due to improper wing drying. Since these adults were immobile, the date of their emergence could be confidently assigned to the corresponding deformed pupa in its place. Butterflies were subsequently placed into glassine envelopes with wings folded behind the ventral edge of the body and on either side of the abdomen, were then euthanized by freezing and stored in a desiccation chamber. Once all trials were completed and adult specimens had been desiccated to a stable dry mass, they were weighed for adult biomass.

Larval death was determined if larvae were unresponsive to physical stimulus such as light prodding with a clean paintbrush and unable to maintain surface attachment. If a specimen began chrysalis formation but was unable to complete chrysalis development to the extent emergence was biologically impossible, the specimen was determined to have died in the pupal stage. In this scenario, date of chrysalis formation and pupal death would be the same. If the specimen did form a functional chrysalis but did not emerge more than two weeks after the last observed emergence for its treatment group, it was also considered a pupal death. Adults with severely deformed or folded wings and that were unable to fly were considered adult deaths and excluded from adult biomass analysis. The mass of deformed adults tended to be much higher than normal adults because they retain meconium that is normally expelled from healthy adults during eclosure.

### Statistical analysis

Linear mixed models with correlated random slopes and intercepts were run for each continuous trait (time to pupation, time to emergence, and adult dry mass). Generalized linear mixed models with random slopes and intercepts were run for binary traits (hatching failure, and cumulative mortality for larva, pupa, and adults) using a binomial distribution with a logit link. For generalized linear mixed effects models, correlations between random slopes and intercepts were unidentifiable, so these terms were fit as uncorrelated. All mixed effects models were run in the R package lme4 (Bates et al. 2014). For all traits, temperature (categorical), terpene concentration (continuous), and their interaction were all treated as fixed effects, whereas the temporally replicated trial run was treated as a random effect. With trial run as the grouping variable, concentration was modeled as a random slope to account for possible heterogeneity across runs. Fixed effects were resampled 10,000 times using the mvrnorm function in the MASS package (Venables and Ripley 2002) to compute 95% bootstrap confidence intervals for LC50s and regression coefficients. For linear mixed models, we visually assessed assumptions using the *performance* package (Lüdecke et al. 2021) to check normality of residuals and random effects, linearity, homogeneity of variance, and leverage. For generalized linear mixed models, we assessed model adequacy by assessing posterior predictive performance. For all models presented we did not observe any notable violations of assumptions. To generate figures, regression lines were plotted using the fixed effects estimates along a grid of predictor values, with the bounds of 95% confidence bands generated by extracting the fixed effects and their covariances for resampling. The median lethal concentration (LC50), represented as percent weight of terpene per total weight of diet (w/w %), was compared at all life stages (Table 1) and used as a metric of relative toxicity where a high LC50 represent low toxicity and a low LC50 represents high toxicity.

**Table 1:** Median lethal concentrations (lc50s) of beta-caryophyllene, cineole, limonene, and linalool during egg hatching, larval, pupal, and adult stages of development at 24°c, 27°c, and 30°c, presented in units of diet concentration on a mass basis (w/w %) as prepared at the start of each trial

		24°C	27°C	30°C
Beta-caryophyllene	Egg	n/a	n/a	n/a
	Larval	1.31 (0.88-2.27)	2.77 (1.64-7.74)	1.21 (0.75-2.04)
	Pupal	0.59 (0.20-1.12)	1.43 (0.54-4.32)	0.38 (0.0-0.79)
	Adult	0.20 (0.0-1.12)	0.07 (0.00-1.62)	0.10 (0.0-0.33)
Cineole	Egg	0.47 (0.36-0.60)	0.53 (0.36-0.71)	2.15 (1.77-2.62)
	Larval	5.62 (2.92-23.46)	4.84 (2.28-22.85)	3.47 (1.81-9.98)
	Pupal	2.99 (1.76-8.83)	2.72 (0.0-17.37)	0.69 (0.42-1.03)
	Adult	0.80 (0.0-2.39)	0.42 (0.0-1.15)	0.30 (0.16-0.44)
Limonene	Egg	1.19 (0.0-1.60)	7.93 (7.56-9.28)	4.93 (2.58-19.62)
	Larval	7.0 (0.0-11.06)	1.63 (0.70-3.15)	2.37 (0.80-7.2)
	Pupal	1.94 (0.0-29.07)	0.43 (0.31-0.57)	0.61 (0.0-2.74)
	Adult	0.72 (0.33-2.56)	0.24 (0.16-0.33)	0.43 (0.03-1.08)
Linalool	Egg	0.06 (0.04-0.08)	0.33 (0.17-0.53)	1.71 (0.0-12.96)
	Larval	2.07 (1.40-3.22)	1.73 (1.19-2.82)	0.86 (0.57-1.56)
	Pupal	1.29 (0.91-1.89)	0.81 (0.60-1.20)	0.35 (0.22-0.56)
	Adult	0.48 (0.26-0.76)	0.23 (0.15-0.33)	0.10 (0.05-0.18)

95% confidence intervals around LC50 estimates are presented in parentheses. ‘NA’ reflects unidentifiable LD50s for data with low hatching failure where logistic regressions failed to converge.

## Results

### Terpene toxicity

All terpenes tested caused an increase in hatching failure at higher concentrations except for beta-caryophyllene (Supplementary Fig. S1) which displayed irregular patterns of hatching inhibition at variable temperatures and concentrations. Beta-caryophyllene overall had the least influence on hatching, with inhibition rarely exceeding 50% at any concentration. Cineole displayed a significantly higher LC50 (i.e., non-overlapping 95% confidence intervals) at 30°C compared to the LC50 observed at 24°C or 27°C, but no difference was observed between 24°C and 27°C (Table 1; Supplementary Fig. S1), suggesting the efficacy of cineole to inhibit hatching decreases at higher temperatures and is greatest at low temperatures. Similarly, limonene and linalool both inhibited hatching most at the lowest temperature (24°C), with significantly lower LC50s than at higher temperature (Table 1, Supplementary Fig. S1). These results suggest that terpenes with roles in hatching inhibition are more powerful at lower temperatures.

The toxicity of the four terpenes in the larval stage were not statistically significantly different by temperature, but the estimated LC50 of both cineole and linalool showed a consistent decrease with increasing temperatures (Table 1, Supplementary Fig. S2). Limonene also had the highest LC50 at the lowest temperature but did not display the same consistent decrease with temperature as linalool and cineole. The sesquiterpene beta-caryophyllene demonstrates no significant differences in LC50 with temperature, nor any substantial trend. Opposite of the patterns observed for hatching failure, the three monoterpenes seem to be more toxic towards larvae at higher temperatures, although not significantly different.

Greater monoterpene toxicity at high temperatures continues in the pupal stage with mortality rates becoming more distinct. Linalool had a significantly lower LC50 at 30°C than both 24 and 27°C (Table 1, Supplementary Fig. S3). The LC50 for cineole were also significantly lower at 30°C than 24°C, with 27°C intermediate. The toxicity of beta-caryophyllene and limonene for the pupal stage did not vary significantly with temperature (Table 1, Supplementary Fig. S3), but the overall trend observed in larva for monoterpenes to have higher toxicity at higher temperatures continued in the pupa stage.

**Fig. 1:**
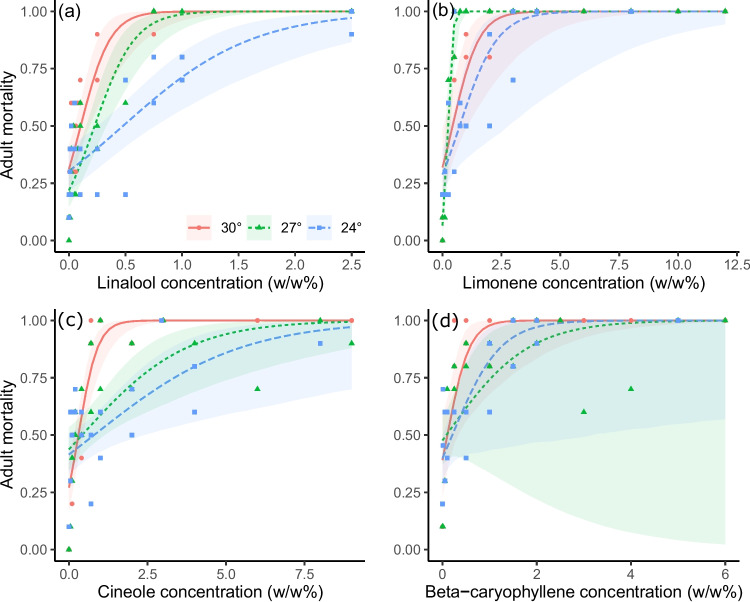
Concentration-response curves of adult mortality at 24°C, 27°C, and 30°C with *V. cardui* subject to varying concentrations of **A**) limonene, **B**) linalool, **C**) cineole, and D) beta-caryophyllene as prepared at the start of each trial. Shaded regions correspond to 95% confidence intervals around each curve. Individual points represent the proportion of adult mortality observed across replicate insects within each concentration for each trial. See Data S1 for full data

In general, adult stage mortality followed the same pattern as larval and pupal mortality, though 95% confidence intervals for LC50s of adult mortality typically overlapped across temperatures for limonene, cineole, and beta-caryophyllene (Table 1, Fig. 1). For linalool, the LC50s for 24°C and 30°C were significantly different, whereas the LC50 for 27°C was intermediate and not significantly different from either 24°C or 30°C (Table 1). The toxicity of linalool and cineole displayed the same trend as for larval and pupal mortality, consistently increasing from 24°C to 27°C to 30°C. However, the other two terpenes differed, with limonene having the lowest adult LC50 and greatest toxicity at 27°C (significantly more toxic at 27°C than 24°C), with 30°C intermediate (Table 1).

### Developmental timing and adult body mass

The number of days to pupation and emergence was plotted against the dietary concentration of each terpene. The individual effect of temperature without the influence of terpenes can be evaluated by comparing y-intercepts (Table 2). Within a given temperature, 95% confidence intervals for y-intercepts for a given developmental event (pupation, emergence) overlapped in all but a single case (cineole, time to pupation at 27°C). This provides context that estimates of development time in non-treated controls were largely consistent across the entire experiment. Comparing y-intercepts across temperatures indicates that higher temperatures speed development, reducing the number of days to both pupation and emergence (Table 2). In all but one case, days to both pupation and emergence were significantly lower at 27°C than 24°C, with a similar magnitude of around 4-5 days (Table 2). Days to pupation and emergence were significantly lower at 30°C than 27°C in half of cases, with a typical magnitude of around 1-4 days regardless of statistical significance (Table 2). In all cases, development time at 30°C was significantly faster than 24°C, with time to development points (e.g. chrysalis formation and adult eclosure) being reached approximately six to eight days faster (Table 2). The magnitude of difference was roughly the same for both time to pupation and time to emergence, indicating that the increased pace of development occurred primarily in the larval stage while specimens were feeding.

**Table 2:** Slopes (Β) and Y-intercepts (Y-int.) of developmental timing (time to pupation, time to emergence) versus concentration at 24°C, 27°C, and 30°C for beta-caryophyllene, cineole, limonene, linalool

			24**°**C	27**°**C	30**°**C
Beta-caryophyllene	Pupation	β	4.10 (2.38-5.88)	2.49 (0.97-4.03)	3.03 (1.35-4.74)
		y-int.	18.77 (17.60-19.95)	13.87 (12.67-15.07)	11.73 (10.37-13.05)
	Emergence	β	3.28 (2.25-4.30)	0.50 (-0.24–1.22)	0.78 (-0.12-1.77)
		y-int.	28.20 (26.87-29.52)	23.59 (22.31-24.89)	20.88 (19.49-22.27)
Cineole	Pupation	β	0.87 (-0.45-2.15)	1.46 (0.15-2.76)	0.10 (-1.18-1.39)
		y-int.	18.99 (17.63-20.36)	16.75 (15.38 -18.14)	11.19 (9.86-12.61)
	Emergence	β	0.39 (-0.28-1.08)	0.84 (0.17-1.50)	-0.18 (-1.03-0.69)
		y-int.	28.14 (26.62-29.71)	24.04 (22.50-25.65)	19.71 (18.14-21.30)
Limonene	Pupation	β	0.80 (0.08-1.73)	3.89 (2.79-4.94)	1.03 (0.03-2.02)
		y-int.	18.43 (16.79-20.03)	13.30 (11.61-15.05)	12.57 (10.80-14.33)
	Emergence	β	0.55 (-2.53-3.79)	6.35 (2.85-9.80)	0.31 (-2.84-3.50)
		y-int.	27.33 (25.77-28.96)	23.51 (21.84-25.21)	21.34 (19.68-23.0)
Linalool	Pupation	β	3.03 (-0.92-7.14)	9.00 (4.82-13.24)	2.07 (-2.32-6.49)
		y-int.	18.68 (17.27-20.09)	14.84 (13.42-16.26)	11.88 (10.49-13.26)
	Emergence	β	3.03 (-2.28-8.41)	9.91 (4.56-15.37)	3.29 (-2.22-8.85)
		y-int.	27.56 (25.88-29.23)	22.68 (20.98-24.34)	20.22 (18.57-21.90)

95% confidence intervals for parameter estimates in parentheses. Slopes reflect the estimated change in developmental timing attributable to increasing terpene concentrations, where a slope of 1.0 is equivalent to an increase in development time of one day per one percent increase in diet concentration (w/w %) as prepared at the start of each trial. Y-intercepts reflect the estimated developmental timing in the absence of terpenes, which is uniformly observed to quicken with increasing temperature.

**Fig. 2:**
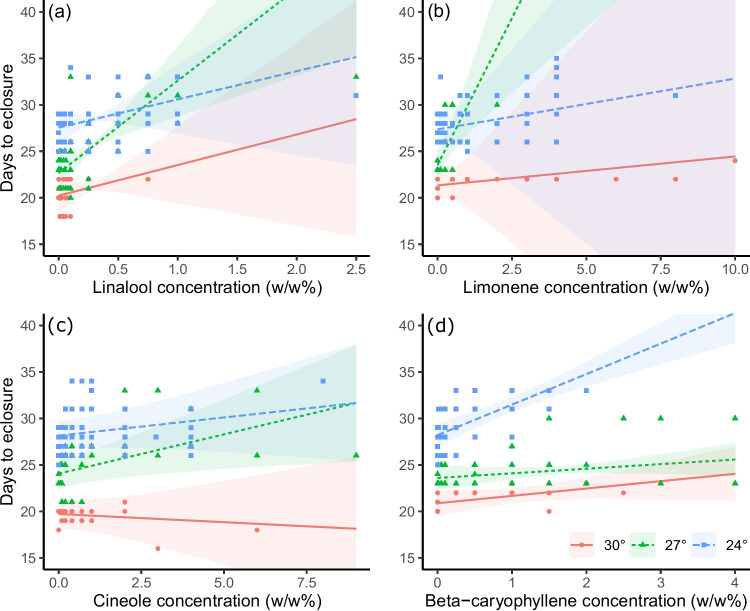
Concentration versus days to pupation at 24°C, 27°C, and 30°C with *V. cardui* subject to varying concentrations of **A**) limonene, **B**) linalool, **C**) cineole, and **D**) beta-caryophyllene as prepared at the start of each trial. Shaded regions correspond to 95% confidence intervals around each curve. Individual points represent the time to pupation observed for each insect that reached pupation within each trial. See Data S2 for full data

Slopes of the relationship between dietary terpene concentration and days to pupation or emergence were compared to detect interactive effects between concentration and temperature. A positive slope that differs from zero indicates that increasing terpene concentration has a delaying effect on development. Further, a significant difference (nonoverlapping confidence intervals) between the slopes observed at two temperatures would indicate that this delaying effect changes with temperature. Time to pupation was observed to have a nonzero slope at all temperatures for beta-caryophyllene and limonene, and at the developmental thermal optimum of 27°C for cineole and linalool (Table 2). The magnitude of these delaying effects varies from roughly 1-9 days per unit terpene concentration (w/w %). Comparison of slopes for time to pupation across temperatures indicates that the magnitude of the delaying effect does not significantly change with temperature for beta-caryophyllene, cineole, or linalool, though is substantially stronger for limonene at the thermal optimum of 27°C than at other temperatures (Table 2). Estimates of slopes for time to emergence were observed to have wide confidence intervals due to smaller sample size (only a subset of individual reach adulthood due to mortality), yet significantly nonzero slopes were observed for cineole, limonene, and linalool at the thermal optimum of 27°C, and for beta-caryophyllene at 24°C (Table 2). Comparisons of slopes for time to emergence find no significant differences with temperature, other than a stronger delaying effect for beta-caryophyllene at 24°C than at higher temperatures (Table 2, Fig. 2).

Generally, adult body mass somewhat declined with increasing dietary terpene concentrations, but effects of temperature and terpene concentration were nonsignificant other than for limonene where the slope of the effect of terpene concentration on adult body mass was significant and negative for 24°C and 30°C (Supplementary Fig. S5). In effect, if *V. cardui* was able to successfully emerge from a chrysalis without visible deformation, average adult size varied within around a two-fold range.

### Limitations to study design

The design choice to replace eggs that failed to hatch with newly hatched young larvae was made in order to enable assessment of post-hatching mortality and developmental timing across the life cycle. Our intent was to emulate oviposition onto a leaf and subsequent insect development on a host plant, and replacement upon hatching failure was conceptually considered to be analogous to a larvae crawling onto a focal leaf from adjacent vegetation. While this design choice maintains replication, it may introduce noise into estimates of both LC50 and development time (increasing variance and the size of confidence intervals) due to the fact that directly hatching insects were exposed to volatile terpenoids while eggs, and replaced larvae were not. However, in treatments where substantial replacement was needed due to hatching failure, mortality for replaced larvae was typically high due to the high terpenoid concentrations present (Dataset S1). Replacement was present across all trials and temperature treatments to a similar extent (34%-48% of larvae across the six trials). We interpret these patterns to suggest that the larval period spent feeding on and touching the diet media was more determinative of post-hatching insect outcomes than pre-hatching volatile exposure to terpenoids, but acknowledge this caveat.

## Discussion

Terpenes are a common and diverse group of plant secondary metabolites with well-established roles in plant defense (Mithofer and Boland 2012; Padovan et al. 2014). Chemical defense compounds can vary in both abundance and composition in plant tissues depending on a wide range of abiotic factors, including temperature (Bidart-Bouzat and Imeh-Nathaniel 2008), which is also the most influential factor driving the pace of insect development (Lehmann et al. 2020). This study explored the potential for common plant terpenes to inhibit the development and survival of a model generalist herbivore, *Vanessa cardui*. Our results show that the effects of common terpenes as an anti-herbivore defense vary with temperature and differentially impact different stages of insect development.

The three monoterpenes included in this study (cineole, limonene, and linalool) all displayed a profound ability to prevent eggs from hatching into larvae, an obviously valuable protective effect that would prevent a host plant from being subject to herbivore feeding damage. Egg hatching inhibition increased with dietary terpene concentrations at all temperatures, but this effect was greatest at lower temperatures. Lepidopteran egg deposition has been shown to induce a number of plant defenses responses, including terpene production (Bertea et al. 2020). Our results support existing evidence that terpene concentrations can have a considerable influence on egg hatching (Hilker and Meiners 2011), yet the mechanism for reduced efficacy of the three monoterpenes under increasing temperature is unclear. Given the lack of direct contact between diet and egg, toxicity must be mediated by diffusion of volatiles to the egg surface, a so-called “fumigation effect” reported in several studies (Chaubey 2012). While diffusion into the egg-adjacent air would be expected to increase with temperature and increase the fumigation effect, increasing temperature would also be expected to increase insect metabolism and therefore perhaps the ability of hatching eggs to detoxify terpenes (Lee et al. 2003).

The impact of temperature on the efficacy of monoterpene defenses switches direction in stages beyond egg hatching, with monoterpenes typically displaying higher toxicity to larvae, pupae, and adults at higher temperatures. Linalool displayed the clearest example of this effect, consistently more toxic at higher temperatures across stages from larvae to adult and statistically significant for cumulative adult mortality. The other two monoterpenes (cineole and limonene) show overall the same temperature pattern in toxicity across the three post-hatching life stages, though only certain temperature comparisons for the pupa and adult stages reach statistical significance. However, the sesquiterpene beta-caryophyllene showed no overall effect of temperature on toxicity across any stage, but given that this was the only sesquiterpene tested we cannot draw broad conclusions for this class. Overall, the opposite effects of temperature on egg hatching versus later stage mortality implies that herbivore susceptibility to monoterpene defenses is highly context-dependent, driven by herbivore life stage, ambient temperature, and compound identity.

Similarly to the toxic effects of terpenes, temperature and concentration had distinct and interactive effects on the developmental timing of specimens. As expected for a poikilothermic ectotherm, the pace of development was faster at higher temperatures. However, each terpene had a differential impact on the pace of development between treatments and concentrations. Most terpenes had nonzero delaying effects observed in at least some temperatures, most commonly at the 27°C thermal optimum for development in *V. cardui*. One mechanism by which terpenes defend plants may be through this extension of development time. Accelerated development time under higher temperatures creates an opportunity for herbivores to both feed more rapidly and reproduce more quickly, and shorter generation time would be expected to increase population size. Both of these effects would escalate the amount of tissue damage experienced by host plants. By delaying development, terpenes may counteract the escalation of herbivore pressure at higher temperatures. Of particular note, even terpenes that exhibited the same absolute magnitude of developmental delaying effect in different temperatures (nondifferent slopes between terpene concentration and development time, Table 2) should properly be considered to have a temperature-dependent delaying effect. This is because the magnitude of delay attributable to terpenes (e.g., 3 days per w/w%) represents a much larger proportion of the total insect development time at warmer temperatures (e.g., 19 days to pupation at 24°C versus 12 days at 30°C), meaning that the same terpene dose inhibits development substantially more at higher temperatures relative to lower temperatures. This may mean that the terpene ‘delaying effect’ is a stronger brake on development at warmer temperatures.

Many plant species have been shown to have seasonal variation in defense investment (Kasey 2010; Karolewski et al. 2013; Mason et al. 2020). Increased production of monoterpenes and sesquiterpenes have been observed to occur in spring and summer which could be a response to either biotic or abiotic conditions (Amaral et al. 2015). We here find that terpenes can be more toxic towards a generalist herbivore at higher temperatures, which suggests during warm seasons plants may be dually defended by increased potency of chemical defenses and their increased production. Variable terpene toxicity was observed across different temperatures, which supports the hypothesis that temperature does impact the efficacy of terpenes as a chemical defense. The shifting of maximum terpene toxicity at different temperatures suggests the existence of terpene production optimums at specific temperatures that would achieve maximum defense against herbivores. All three monoterpenes were less toxic at lower temperatures, so plants would have to produce substantially higher concentrations in colder seasons or climates in order to achieve the same level of defense provided at warmer temperatures. However, herbivore pressure by poikilothermic ectotherms like insects is known to be lower during cooler seasons, such that seasonal trends in terpene production may track herbivore feeding activity either through chemical defense induction (Karban 2011; Züst and Anurag 2017) or seasonal shifts in defense expression arising from either seasonal changes in resource availability or pre-programmed ontogenetic or environmental responses (Schönwitz et al. 1990; Barton and Koricheva 2010; Mason et al. 2020). Terpene production in plants has generally been found to increase under higher temperatures (Ibrahim et al. 2010; Yang et al. 2021), which could mean that plants are able to increase resource allocation to defense under favorable environmental conditions like those typically found during the peak of the growing season (Zvereva and Kozlov 2006), or perhaps that higher terpene production in warmer seasons is a pre-programmed response shaped by natural selection to counteract faster herbivore development.

Plant nutritional quality (as indicated by C:N ratio) has been shown to decrease under elevated CO_2_ levels at both ambient and elevated temperatures, a phenomenon known as the carbon fertilization effect (Lawler et al. 1996; Drake et al. 2017; Davis et al. 2022). This reduced nutritional quality stimulates herbivores to consume more plant tissue in order to reach their minimum nutritional needs, a phenomenon known as compensatory feeding (Simpson and Simpson 2017). However, no significant fluctuations in nutritional quality have been observed under increased temperatures at ambient CO_2_ levels (Soares et al. 2019). Meta-analysis indicates that terpene production is largely unaffected by CO_2_ elevation alone, but is increased when temperature and CO_2_ are elevated simultaneously (Zvereva and Kozlov 2006)_._ It is possible the combined effect of low nutritional quality and increased terpene production may be enough to counteract the accelerated development of insect herbivores at high temperatures, though this will require careful examination given species- and habitat-specific effects. How future climatic conditions will impact plant responses to herbivory remains largely uncertain at this time, but our results in the context of existing literature indicate that shifts in the production and efficacy of major classes of plant chemical defenses like terpenoids are likely. Previous work indicates that terpene production will likely increase (Holopainen and Gershenzon 2010), and this study suggests that terpene toxicity and developmental effects on herbivores may change as well. If increasing terpenoid production and efficacy imposes stronger selection for insect resistance through detoxification and other mechanisms, herbivore populations may evolve higher tolerance to plant defenses in habitats experiencing substantial warming.

Given that over 40% of global crop production is lost to insect pests annually (IPPC 2021), the finding that plant terpenoids may be more effective (lower LC50) against insect pests at higher temperatures would seem to bode well for agricultural productivity in the face of climate change. However, the benefits of increased terpene toxicity may be counteracted by increased pest voltinism if warming temperatures lengthen the active season while simultaneously speeding insect maturation (Forrest 2016). Plant chemical defenses may be able to slow pest development and interfere with survival to adulthood, but our results suggest that terpene-induced developmental delays are weaker at higher temperatures. Given that insects are expected to experience compensatory feeding as increased warming and atmospheric CO_2_ increase leaf C:N ratio, this suggests that a given insect pest will consume more plant tissue to meet its dietary needs. Furthermore, increased voltinism equates to an increase in the number of generations per unit absolute time, which increases the potential rate of evolution of populations (Maino et al. 2018). Increased voltinism may also provide the potential for increased insect population size, especially for migratory species that re-establish in a region each active season (Buckley et al. 2017). Increasing population sizes and increased rate of evolution provide increased capacity for insect populations to adapt to new selective pressures (Lande 1988; Debarre and Gandon 2011; Gravel 2016; Jensen et al. 2017), including plant chemical defenses or synthetic pesticides.

**Fig. 3:**
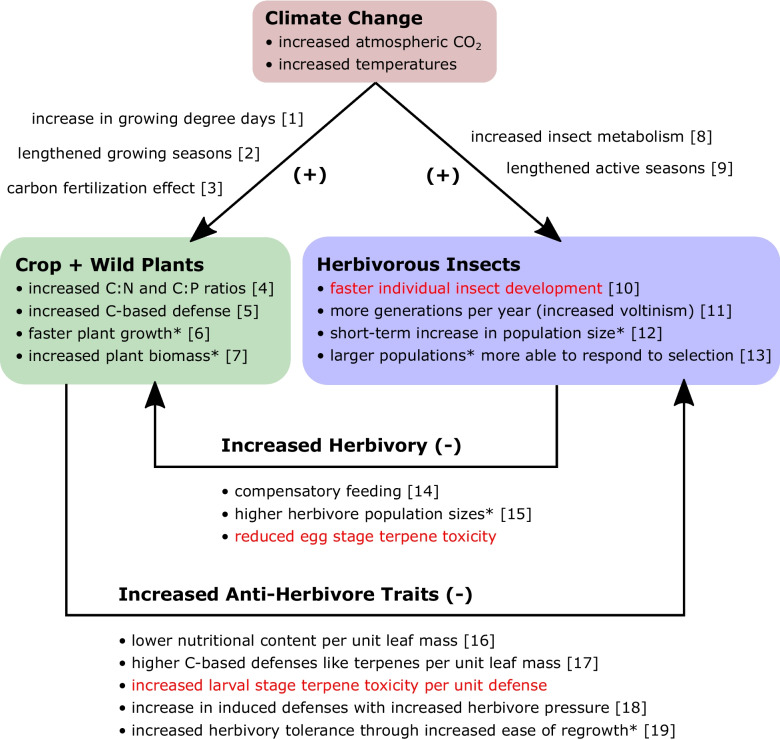
Broader implications of temperature-dependent terpene toxicity under climate change should the results observed for the polyphagous *Vanessa cardui* in this study (red text) hold for other herbivorous insects. Increased temperatures and atmospheric carbon dioxide concentrations observed under climate change are predicted to have multiple cascading effects on both plants and herbivorous insects. For plants, the increase in growing degree days [1], lengthened growing seasons [2], and the carbon fertilization effect [3] have been demonstrated to result in increased leaf C:N and C:P ratios [4] and increased carbon-based plant defenses [5]. In addition, where other factors like water and nutrient availability are not limiting (*), faster plant growth per unit time [6] is expected to result in increased plant biomass production [7]. For insects, the increase in metabolic rate with increased temperature [8] and the lengthening of active seasons [9] have been demonstrated to result in faster individual development [10] as observed in this study, as well as an increase in the number of generations per year [11], including shifts from univoltine to multivoltine life history in a given geographic region. Where other factors do not limit insect population growth (*), faster development and shorter generation time are expected to result in short-term increases in population size [12], such as within a single growing season. Larger populations of organisms are known to be more able to respond to natural selection [13]. The interactions between plant and insect responses are expected to have multiple effects that influence both the magnitude of herbivory and anti-herbivore plant traits. For insects, the phenomenon of compensatory feeding [14] due to lower leaf nutrient content combined with higher herbivore population sizes [15] would be expected to result in an increase in herbivory pressure on plants. This could be further heightened if hatching rates are higher due to reduced egg stage terpene toxicity as observed in this study, regardless of whether larvae survive to reproduction. For plants, lower nutritional content per unit leaf mass has been demonstrated to reduce herbivore survival and development [16], as have higher concentrations of carbon-based defenses (like terpenes) per unit leaf mass [17]. If the potency of these defenses (toxicity of a given concentration) is also increased by temperature as observed in this study, this will act as a multiplier of the efficacy of plant chemical defense investment. An increase in the magnitude or duration of herbivore pressure would be expected to induce additional production of chemical defenses [18], which may increase concentrations further. Additionally, to the extent that plant growth rate may increase (*) due to the factors described above, this would be expected to increase plant herbivory tolerance through the regrowth of lost or damaged plant parts [19]. Overall, these many individual effects suggest a potential intensification of the plant-herbivore interaction under climate change. *References*: [1+2] Park et al. 2016; Kukal and Irmak 2018a; Matthews et al. 2018; Piao et al. 2019; [3] McGrath and Lobell 2013; Liang et al. 2016; Terrer et al. 2019; Ueyama et al. 2020; [4] Lincoln et al. 1993; Bezemer and Jones 1998; Gifford et al. 2000; Stiling and Cornelissen 2007; Sardans et al. 2012; Wang et al. 2021; [5+17] Filella et al. 2007; Helmig et al. 2007; Bidart-Bouzat and Imeh-Nathaniel 2008; Ibrahim et al. 2010; Cornelissen 2011; [6+7] Gray and Brady 2016; Park et al. 2016; Kukal and Irmak 2018b; Piao et al. 2019; Babst et al. 2019; Ueyama et al. 2020; [8+9] Cayton et al. 2015; Deutsch et al. 2018; Jactel et al. 2019; Forrest 2016; [10] Colinet et al. 2015; Buckley et al. 2017; Rebaudo and Rabhi 2018; Harvey et al. 2020; [11] Tobin et al. 2008; Altermatt 2010; Ziter et al. 2012; Forrest 2016; [12+15] Colinet et al. 2015; Ziska and McConnell 2016; Tonnang et al. 2017; Harvey et al. 2020; Wagner et al. 2021; Schneider et al. 2022; [13] Lande 1988; Debarre and Gandon 2011; Gravel 2016; Jensen et al. 2017; but see Wood et al. 2016; [14+16] Fajer et al. 1989; Stiling and Cornelissen 2007; Trebicki et al. 2017; Hamann et al. 2021; [18] Agrawal 1998; Agrawal et al. 1999; Underwood 2003; Copolovici et al. 2011; [19] Tiffin 2000; Fornoni 2011; Gray and Brady 2016.

Overall, these many individual effects suggest a potential intensification of the plant-herbivore interaction under climate change (Fig. 3). Larger populations of faster-developing insects consume larger amounts of plant tissue, while faster growing plants produce higher quantities of lower-nutrient biomass defended with higher concentrations of more effective carbon-based chemical defenses. While plants might be expected to prevail under such conditions, if large populations of insects are subjected to diets where individuals must ingest larger quantities of more-toxic chemical defenses to meet their minimum nutritional needs, that would constitute a strong selective force for the evolution of resistance to those chemical defenses. While this might result in plants and herbivores reaching a new equilibrium in wild plant populations, this would be unlikely in agricultural settings where crop plants are not typically permitted to evolve in response to herbivore pressure. The evolution of increased insect resistance to plant chemical defenses in natural settings could be a further contributor to agricultural impacts of pest insects, where large increases have been observed thus far under climate change (IPPC 2021). Although this study is limited in scope to the effects of a handful of common plant terpenes in a single cosmopolitan insect herbivore, the patterns of temperature-dependent effects identified raise major questions about the influence of temperature on the efficacy of plant chemical defenses. Temperature-dependent effects of plant chemical defenses should therefore be explicitly considered in the study of seasonality in the fundamental process of herbivory, and the modeling of plant-insect interactions under climate change.

### Supplementary Information

Data 1 Full data on *Vanessa cardui* hatching failure and larval, pupa, and adult mortality presented as counts and proportions. Run represents individual trial runs, and temperature is presented in Celsius. Concentration of each terpene is presented in w/w percentage as prepared at the start of each trial. See full text for methods details. (CSV 12 kb)

Data 2 Full data on *Vanessa cardui* development time from hatching to pupation, and from hatching to eclosure (emergence from pupa). Run represents individual trial runs, and temperature is presented in Celsius. Concentration of each terpene is presented in w/w percentage as prepared at the start of each trial. See full text for methods details. (CSV 39 kb)

Data 3 Full data on *Vanessa cardui* adult dry mass. Run represents individual trial runs, and temperature is presented in Celsius. Concentration of each terpene is presented in w/w percentage as prepared at the start of each trial. See full text for methods details. (CSV 29 kb)

ESM 4 (DOCX 4357 kb)
