# Supplementary material for: Temperature alters the toxicological impacts of plant terpenoids on the polyphagous model herbivore Vanessa cardui
Source: J Chem Ecol. 2023 Sep 11;49(11-12):666–80. doi: 10.1007/s10886-023-01449-8 (PMC10781811; doi:10.1007/s10886-023-01449-8)
Supplement: Supplementary file 4 — (DOCX 4357 kb) [file 10886_2023_1449_MOESM4_ESM.docx]

Supplemental Figures for:


Title: **Temperature alters the toxicological impacts of plant terpenoids on the polyphagous model herbivore *Vanessa cardui***

Authors: Mari R. Irving, Eric W. Goolsby, Hannah Stanford, Simone Lim-Hing, Maria Urrea, Chase M. Mason

Journal: Journal of Chemical Ecology

Corresponding author: Mari R. Irving: mari.ecoevo@gmail.com

Supplemental Figure 1


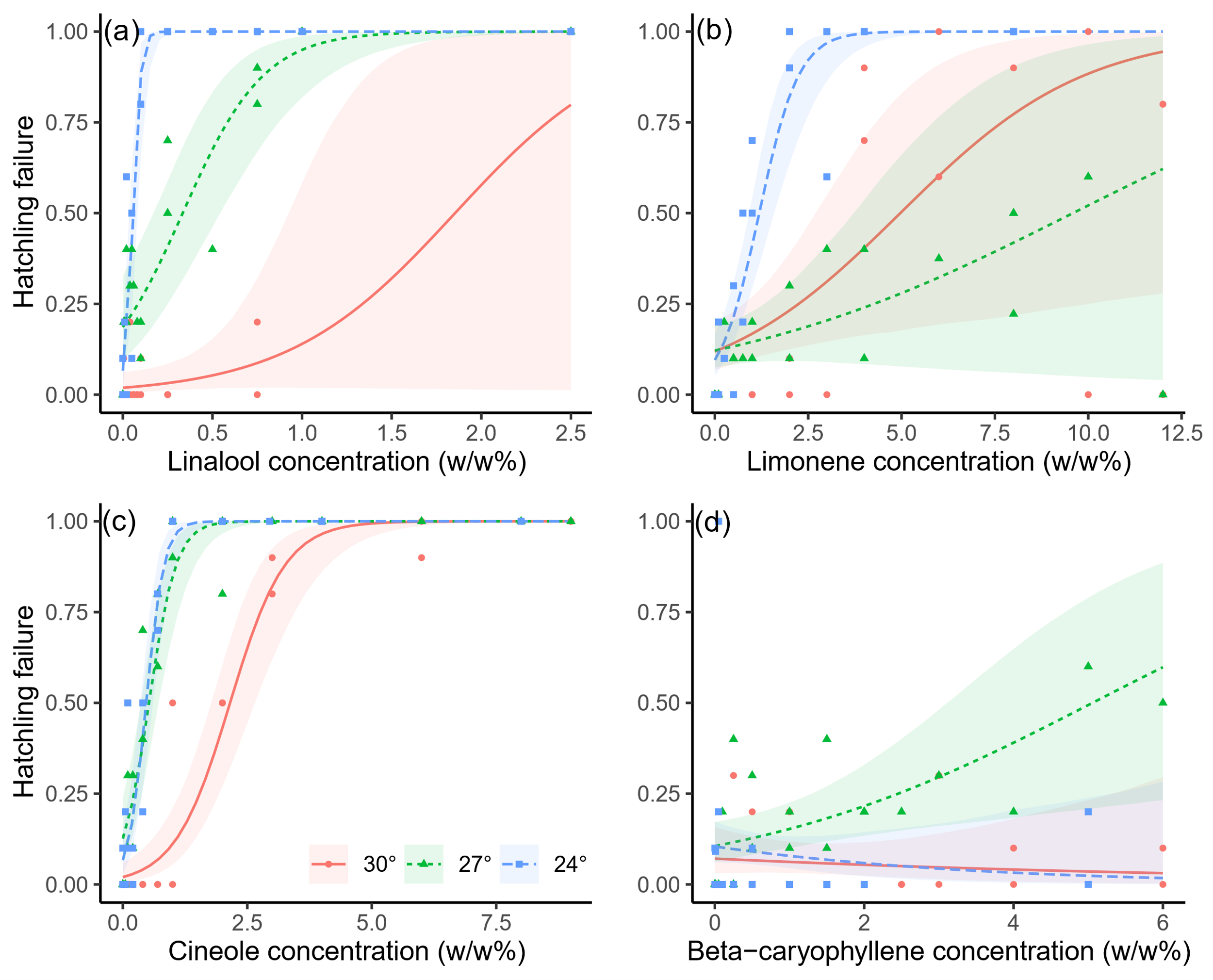


Figure S1. Concentration-response curves of hatching failure at 24°C, 27°C, and 30°C with *V. cardui* eggs subject to varying concentrations of A) limonene, B) linalool, C) cineole, and D) beta-caryophyllene. Shaded regions correspond to 95% confidence intervals. Concentrations refer to w/w% composition of the diet as prepared at the start of each trial. Individual points represent the proportion of hatching failure observed across replicate insects within each concentration for each trial. See Data S1 for full data.

Supplemental Figure 2


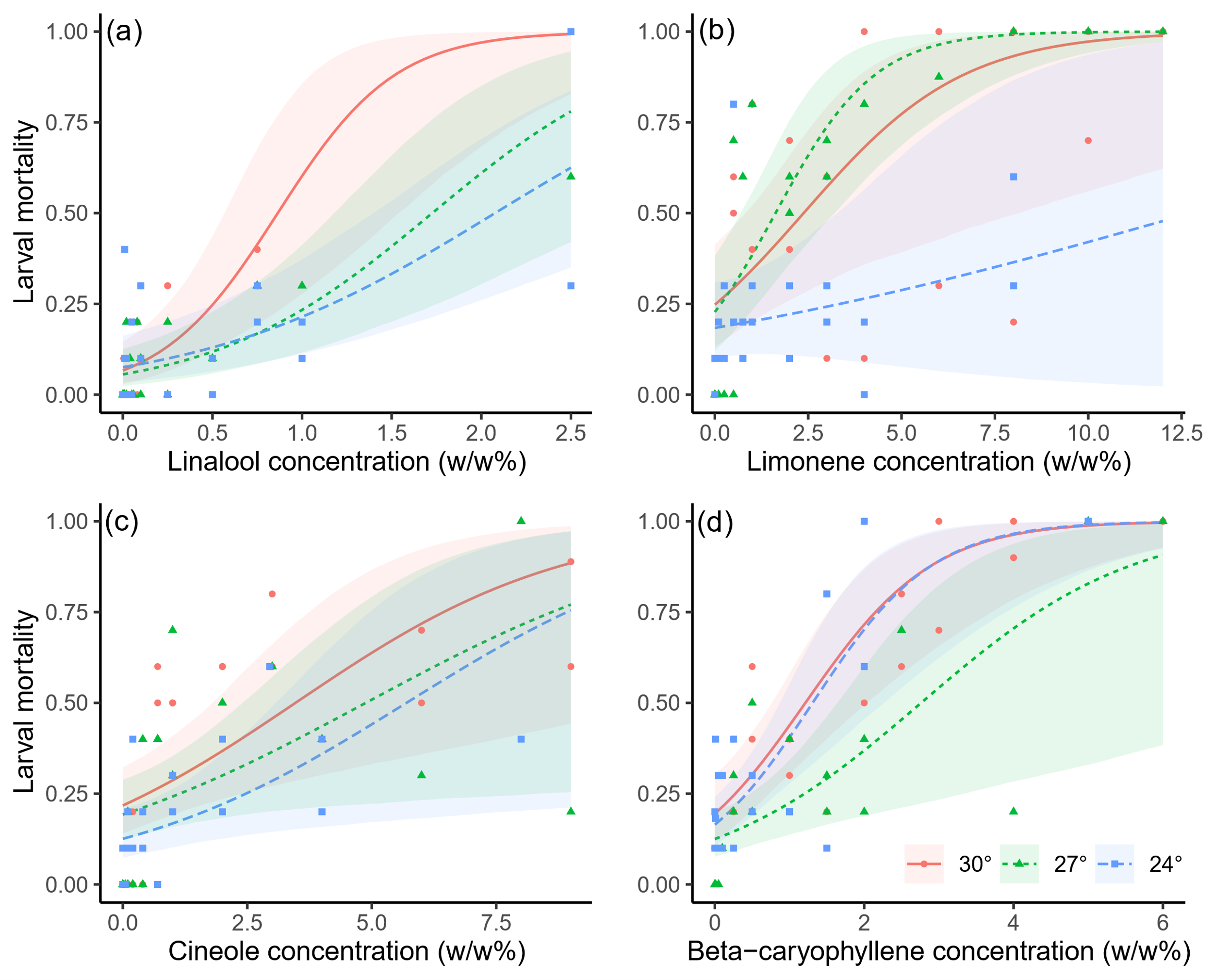


Figure S2. Concentration-response curves of larval mortality at 24°C, 27°C, and 30°C with *V. cardui* subject to varying concentrations of A) limonene, B) linalool, C) cineole, and D) beta-caryophyllene. Shaded regions correspond to 95% confidence intervals. Concentrations refer to w/w% composition of the diet as prepared at the start of each trial. Individual points represent the proportion of larval mortality observed across replicate insects within each concentration for each trial. See Data S1 for full data.

Supplemental Figure 3


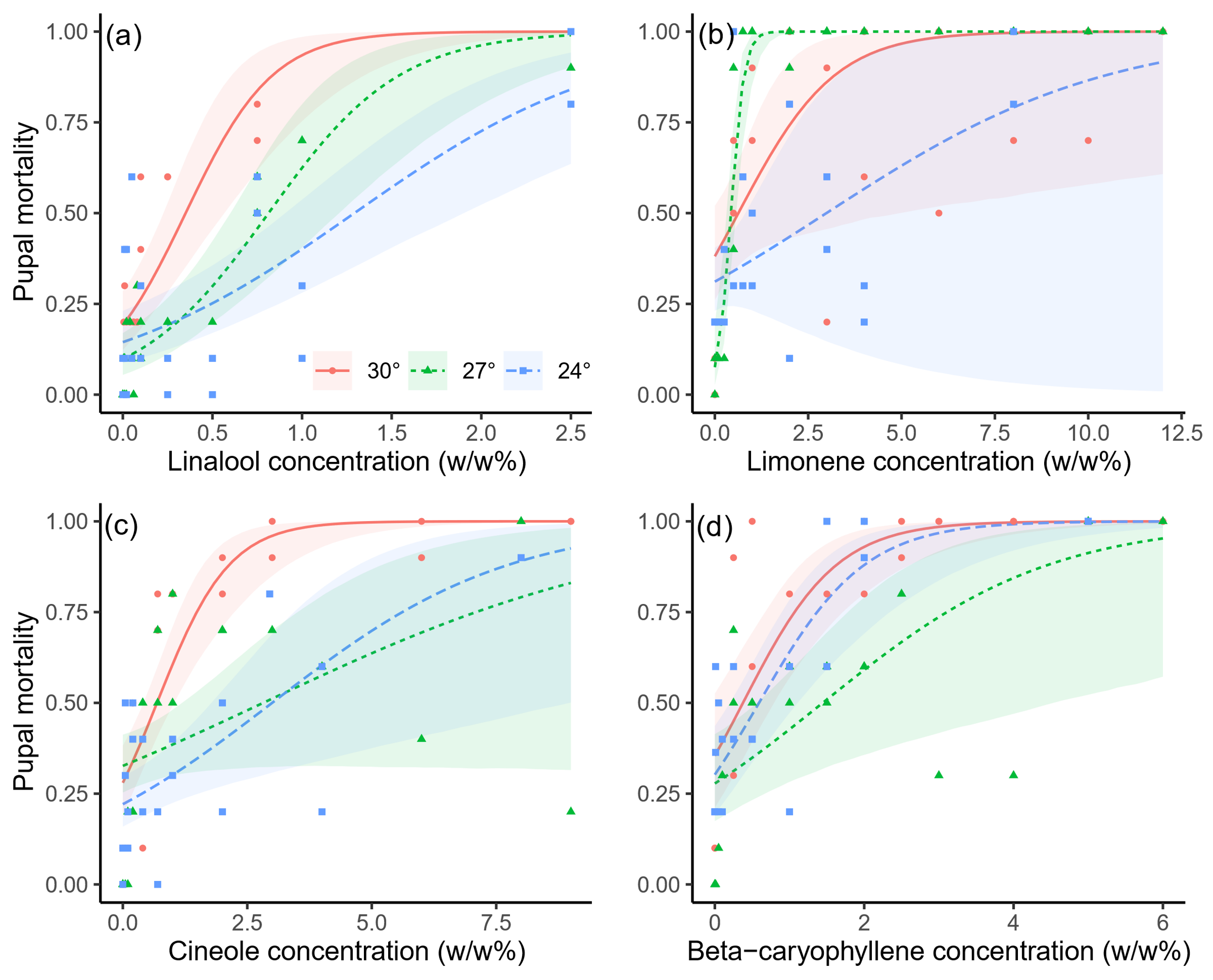


Figure S3. Concentration-response curves of pupal mortality at 24°C, 27°C, and 30°C with *V. cardui* subject to varying concentrations of A) limonene, B) linalool, C) cineole, and D) beta-caryophyllene. Shaded regions correspond to 95% confidence intervals. Concentrations refer to w/w% composition of the diet as prepared at the start of each trial. Individual points represent the proportion of pupal mortality observed across replicate insects within each concentration for each trial. See Data S1 for full data.

Supplemental Figure 4


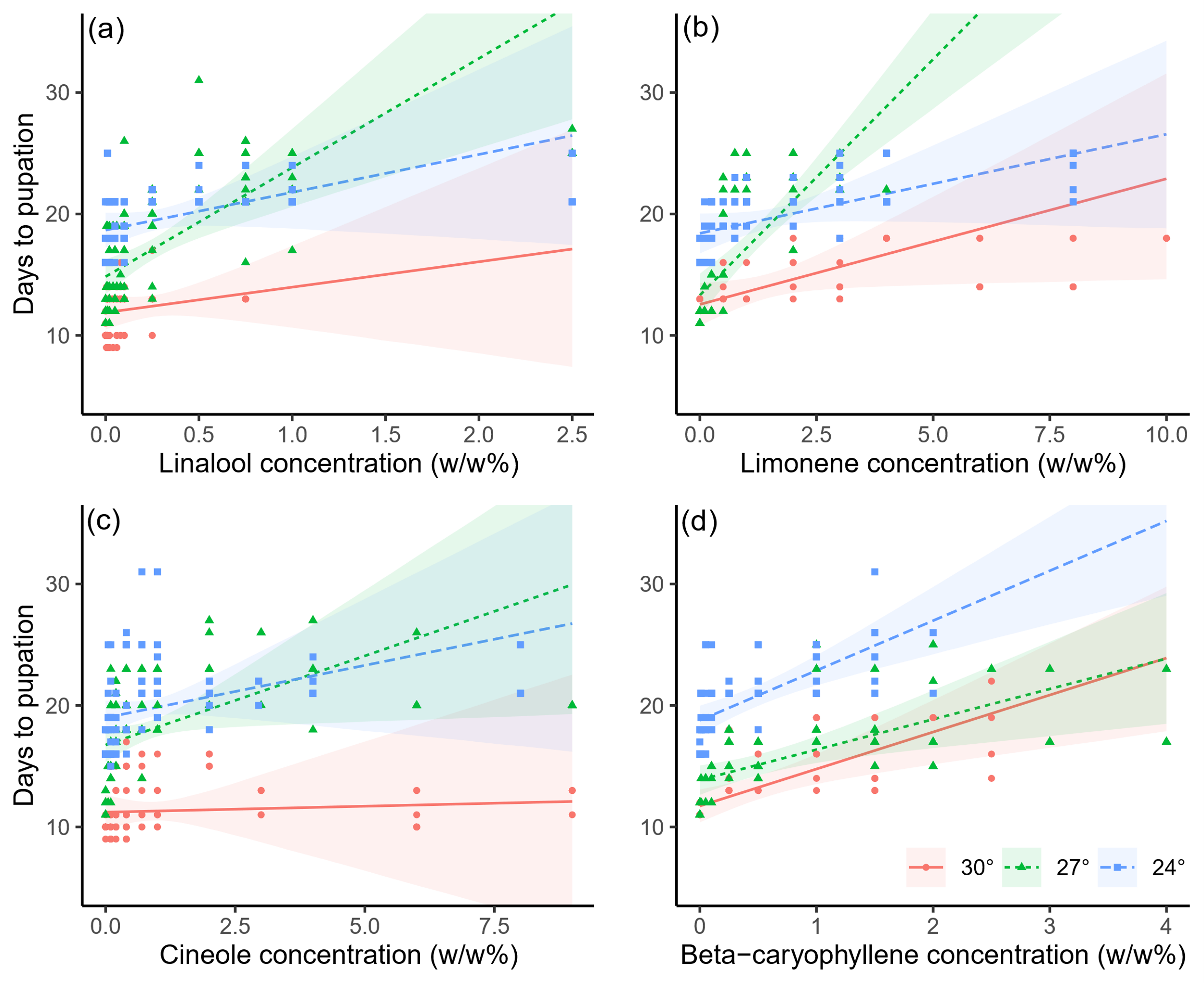


Figure S4. Concentration versus days to pupation at 24°C, 27°C, and 30°C with *V. cardui* subject to varying concentrations of A) limonene, B) linalool, C) cineole, and D) beta-caryophyllene. Shaded regions correspond to 95% confidence intervals. Concentrations refer to w/w% composition of the diet as prepared at the start of each trial. Individual points represent the time to pupation observed for each insect that reached pupation within each trial. See Data S2 for full data.

Supplemental Figure 5


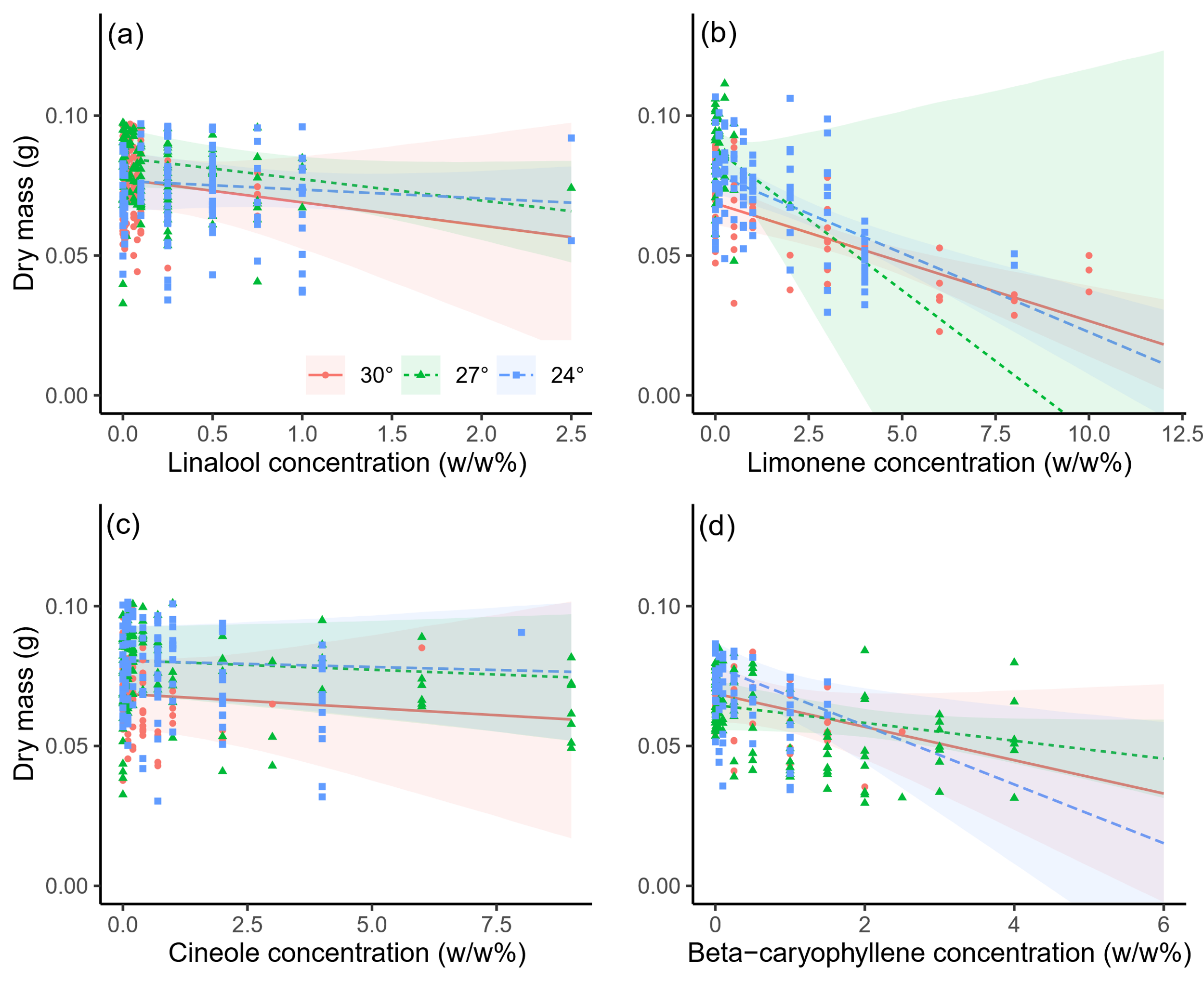


Figure S5. Dry mass of adults reared at 24°C, 27°C, and 30°C with *V. cardui* subject to varying concentrations of A) limonene, B) linalool, C) cineole, and D) beta-caryophyllene. Shaded regions correspond to 95% confidence intervals. Concentrations refer to w/w% composition of the diet as prepared at the start of each trial. Individual points represent the dry biomass observed for each fully-formed adult that eclosed within each trial. See Data S3 for full data.
